# Genetic Population Structure of Wild Pigs in Southern Texas

**DOI:** 10.3390/ani11010168

**Published:** 2021-01-12

**Authors:** Johanna Delgado-Acevedo, Angeline Zamorano, Randy W. DeYoung, Tyler A. Campbell

**Affiliations:** 1 Texas A&M University-Commerce, STC 262, Commerce, TX 75429, USA; 2 Caesar Kleberg Wildlife Research Institute, Texas A&M University-Kingsville, Kingsville, TX 78363, USA; angeline_z00@yahoo.com (A.Z.); randall.deyoung@tamuk.edu (R.W.D.); 3 East Foundation, 200 Concord Plaza Dr., Suite 410, San Antonio, TX 78216, USA; tcampbell@eastfoundation.net

**Keywords:** wild pig, genetic population structure, invasive species, *Sus scrofa*

## Abstract

**Simple Summary:**

Wild pigs are the most abundant wild exotic ungulate in the United States. In Texas, particularly, they are abundant and represent a threat to ecosystems, agriculture and humans. Our objective was to apply a landscape-scale analysis of population genetic structure of wild pigs to aid in their management in southern Texas. We used microsatellites to assist large-scale applied management. We found that some populations were isolated from one another. However, many individuals and local populations were admixed, which indicates that multiple introductions and artificial movement of individuals has occurred. Wild pig management efficiency and effectiveness may be able to improve if illegal translocations stop (e.g., enforcing laws) and if management cooperatives are created to manage spatially extensive areas of southern Texas.

**Abstract:**

Wild pigs (*Sus scrofa*) alter ecosystems, affect the economy, and carry diseases that can be transmitted to livestock, humans, and wildlife. Understanding wild pig movements and population structure data, including natural population boundaries and dispersal, may potentially increase the efficiency and effectiveness of management actions. We trapped, conducted aerial shootings, and hunted wild pigs from 2005 to 2009 in southern Texas. We used microsatellites to assist large-scale applied management. We quantify broad-scale population structure among 24 sites across southern Texas by computing an overall F_ST_ value, and a Bayesian clustering algorithm both with and without considering the spatial location of samples. At a broad geographic scale, pig populations displayed a moderate degree of genetic structure (F_ST_ = 0.11). The best partition for number of populations, based on 2nd order rate of change of the likelihood distribution, was K = 10 genetic clusters. The spatially explicit Bayesian clustering algorithm produced similar results, with minor differences in designation of admixed sites. We found evidence of past (and possibly ongoing) translocations; many populations were admixed. Our original goal was to identify landscape features, such as barriers or dispersal corridors, that could be used to aid management. Unfortunately, the extensive admixture among clusters made this impossible. This research shows that large-scale management of wild pigs may be necessary to achieve control and ameliorate damages. Reduction or cessation of translocations is necessary to prevent human-mediated dispersion of wild pigs.

## 1. Introduction

Large-scale management of wildlife populations has increased in recent decades in response to invasive species, animal disease, and similar challenges, all of which threaten entire ecosystems and humans. For instance, invasive species may affect ecosystem function by changing the flow of energy and biomass, disrupting disturbance regimes, and changing the physical structure of ecosystems [1,2,3]. Invasive species can affect the availability of nutrients for other species and compete with other species in both space and time. In addition to ecological effects, invasive species may pose a disease risk. Diseases that cross the wildlife, human, and livestock interface have health, economic, and social ramifications over entire geographic regions, as evidenced by highly publicized recent outbreaks of influenza, rabies, bovine tuberculosis, and foot and mouth disease. Wildlife management challenges are expected to increase in coming years through global climatic changes, land-cover and land-use changes resulting from anthropogenic activities, and natural and unnatural movements of pathogens [4,5,6].

Wild pigs (*Sus scrofa*) are distributed through much of the world and have become invasive in most of their range. Wild pigs are the most abundant wild exotic ungulate in the United States. Wild populations in the United States are a mixture of domestic pig, Eurasian wild boar, and the hybrids of these two forms [7]. In a recent survey of wild pig populations, the Animal and Plant Health Inspection Service reported wild pigs occurring in 33 U.S. states, spanning from California to Virginia, with isolated populations further north [8]. Estimates of the total United States population are up to 6.9 million animals by 2016 [9], with as many as 2.6 million occurring in Texas [10]. However, Mellish et al. 2014 [11] reported population sizes increased ranging from 3.6 to 16.9 million in 5 years based on an estimated a mean annual growth rate of 0.32. 

A primary challenge in vertebrate invasive species management is the delineation of management zones. Effective management requires a twofold action: definition of a target area for management and ensuring containment of the managed area. Therefore, practitioners must manage at the scale of local populations, and identify and target dispersal corridors [12,13]. One problem is how to define the target area when there are no obvious breaks or population boundaries and little specific knowledge of animal movements and dispersal in the management area. Animal movements are typically not random across the landscape but are influenced by a variety of environmental and social factors. Management decisions informed by population structure, including natural population boundaries and dispersal corridors (rivers, streams, etc.), dramatically increase the success of management actions. In this manner, management efforts are concentrated at specific sites, thus increasing efficiency and effectiveness of management actions.

Without prior knowledge, management zones are often defined arbitrarily (e.g., according to political boundaries) or with the best available knowledge. Traditional wildlife investigations, involving tagging and radio-telemetry, can provide valuable information on animal movements and dispersal but are time-consuming and limited by constraints on sample size. Accordingly, an increasing number of studies use genetic information to assist large-scale applied management [14,15,16,17,18,19].

Our objective was to apply a landscape-scale analysis of population genetic structure of wild pigs to aid in their management in southern Texas. This region includes agricultural areas where landowners experience significant damage to crops, rangeland ecosystems, and natural resources due to abundant populations of wild pigs. This is also an area where wild pigs are hunted recreationally and live-trapped for commercial pork markets. Understanding how wild pig populations are structured will provide a foundation for development of contingency plans in the event of exotic disease outbreaks and assist in the delineation of management zones to establish more effective management strategies. 

## 2. Materials and Methods

### 2.1. Sample Collection, DNA Extraction, and Amplification

**Figure 1:**
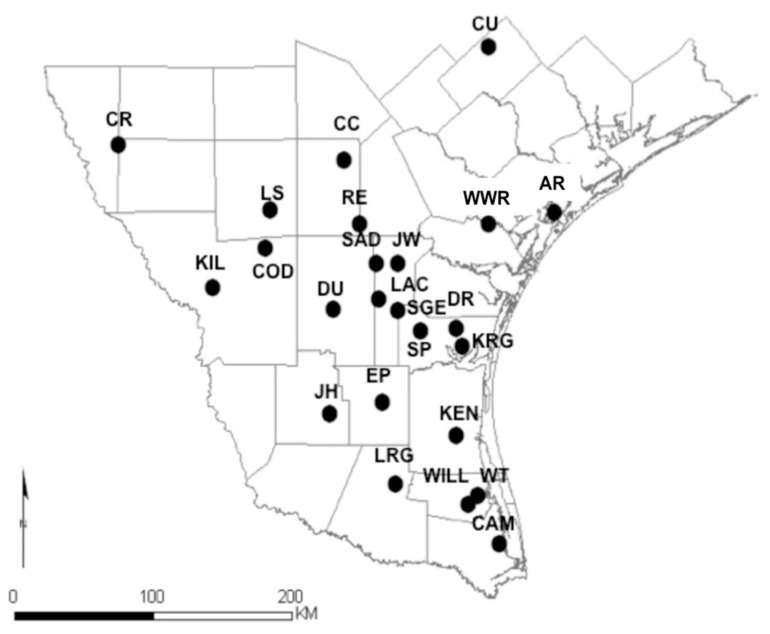
Study sites and sampling locations of tissue samples distributed along southern Texas. Study sites are labeled in the figure. Aransas National Wildlife Refuge (AR), Cameron County (CAM), Choke Canyon State Park (CC), Kubala’s Ranch (COD), Comanche Ranch (CR), Cuero County (CU), Don Ricardo pasture, Laureles Division of King Ranch (DR), Duval County (DU), El Pintor Ranch (EP), Jim Hogg County (JH), Jim Wells County (JW), Kenedy Ranch (KEN), Killam Ranch (KIL), Gallito pasture, Laureles Division of King Ranch (KRG), the Texas A&M Extension Service La Copita Research Area (LAC), Lower Rio Grande Valley National Wildlife Refuge (LRG), La Salle County (LS), Rancho Escondido (RE), San Diego County (SAD), South Pasture-Texas A&M-Kingsville (SP), Willacy County (WILL), Wilbarger Tract, Lower Rio Grande Valley National Wildlife Refuge (WT), Rob and Bessie Welder Wildlife Refuge (WWR), and Santa Gertrudis division of King Ranch (SGE).

We obtained tissue (muscle) samples from wild pigs at 24 sites (Figure 1) throughout southern Texas from 2005–2009: Aransas National Wildlife Refuge (AR), Cameron County (CAM), Choke Canyon State Park (CC), Kubala’s Ranch (COD), Comanche Ranch (CR), Cuero County (CU), Don Ricardo pasture, Laureles Division of King Ranch (DR), Duval County (DU), El Pintor Ranch (EP), Jim Hogg County (JH), Jim Wells County (JW), Kenedy Ranch (KEN), Killam Ranch (KIL), Gallito pasture, Laureles Division of King Ranch (KRG), the Texas A&M Extension Service La Copita Research Area (LAC), Lower Rio Grande Valley National Wildlife Refuge (LRG), La Salle County (LS), Rancho Escondido (RE), San Diego County (SAD), Santa Gertrudis division of King Ranch (SGE), South Pasture, Texas A&M University-Kingsville (SP), Willacy County (WILL), Wilbarger Tract, Lower Rio Grande Valley National Wildlife Refuge (WT), and Rob and Bessie Welder Wildlife Refuge (WWR). We trapped, hunted, aerial gunned, and euthanized animals (as part of population control and eradication efforts) at georeferenced locations within each site prior to tissue collection. We placed tissue samples in 70% ethanol and stored them at −20 °C. We extracted total DNA using a commercial kit (Qiagen DNeasy, Qiagen Genomics, Bothell, Washington, DC, USA). We genotyped 13 microsatellite DNA markers that were designed as part of the Pig Genome Mapping Project. These loci are polymorphic, unlinked, and easy to amplify and score. Marker loci were amplified using the polymerase chain reaction (PCR) [19]. The PCR products were loaded onto an ABI 3130 automated DNA sequencer (Applied Biosystems, Foster City, CA, USA) for separation and detection. We binned and assigned alleles and constructed multilocus genotypes for all individuals using GeneMapper (Applied Biosystems, Foster City, CA, USA). 

### 2.2. Locus Properties and Genetic Differentiation among Populations

We estimated allelic richness [20] and evaluated departures from Hardy–Weinberg equilibrium in FSTAT [21]. We assessed significance of departure from Hardy–Weinberg expectations by 1000 randomizations of alleles among individuals and corrected for multiple comparisons using a Bonferroni procedure [22]. We tested for a relationship between genetic and geographic distance to determine if population structure follows isolation by distance pattern [23]. This is because Bayesian clustering algorithms may overestimate the number of genetic clusters in continuous populations, where genetic structure may be a function of geographic distance among clusters [18]. We performed a Mantel test [24] to assess the correlation between the geographic and genetic distance matrices by 10,000 permutations of rows and columns using the computer program Genepop 3.4 [25]. We quantified the Euclidian geographic distance among all pairs of sampling sites, then computed the pairwise genetic distance across sites using Nei’s Ds [26], which indicates the genetic similarity based on allele frequencies per locus among populations. The Ds values range from 0 to 1, where 0 denotes similar allele frequencies, and 1 denotes no allele sharing. We used SPAGeDi 1.2 [27] to calculate and construct the genetic and geographic distance matrices.

We evaluated genetic structure and differentiation among populations using both fixation statistics and Bayesian clustering methods. We quantified broad-scale population structure among the 24 sites by computing an overall F_ST_ value [28], which measures the differentiation of subpopulations relative to the total sample, as an index of population structure. We employed 2 separate Bayesian clustering algorithms to evaluate population structure, both with and without considering the spatial location of samples. First, we used a Bayesian implementation in the program Structure 2.2 to group individuals into clusters (K) that minimize Hardy–Weinberg and linkage disequilibrium without regard to population of origin [29]. We used a burn-in of 150,000 repetitions, followed by 250,000 MCMC iterations, assuming allele frequencies were correlated. We modeled from K = 1–24 clusters, 10 repetitions of each cluster. We used the ΔK statistic, the second order rate of change of the likelihood distribution [30], to determine the number of genetic clusters in the data set. Admixture proportions (*q*-values) for each individual, based upon MCMC runs where K was set at the best fit, were used to define cluster membership. Individuals were considered to be assigned to a cluster if *q* > 0.8; individuals with *q* < 0.8 were considered admixed.

Second, we also performed a Bayesian clustering analysis that used spatial information and implemented in the program BAPS 4.2 [31]. This Bayesian method characterizes genetically differentiated clusters based on genetic data and geographical location of samples. BASP attempts to identify populations with different allele frequencies, rather than attempting to minimize HWE and linkage disequilibrium, as in Structure 2.2, therefore these two methods are complementary. Stochastic optimization is used in BAPS 4.2 to assume posterior mode of the number of subpopulations, where spatial location of samples and allele frequency divergence among sampling sites are considered [32]. We conducted the spatial clustering analysis, setting the maximum number of clusters at 24; we performed 10 repetitions for each cluster to evaluate consistency among runs. The program reports the probabilities for different numbers of genetic clusters and determines the optimal partition. Stored results based on log-likelihood values in BAPS 4.2 are merged to compute a distance matrix among genetic clusters based on the Kullback–Leibler distance that can be used as a relative measure of genetic divergence between genetic clusters [31]. We constructed a neighbor-joining tree [33] based on the Kullback–Leibler distances from Structure 2.2 and BAPS 4.2 using the computer program Mega 4.0 [34] to visualize similarity among genetic clusters. 

Third, we conducted a Principal Components Analysis (PCA) using the *adegenet* package [35,36] for R software. Principal Components Analysis (PCA) will provide a description of a large number of measurements (e.g., alleles) reducing them to a few dimensions (e.g., clusters) to explain patterns on the data. In addition, we conducted a Discriminant Analysis of Principal Components (DAPC) [35] using the *adegenet* package [36] for R software. Discriminant Analysis of Principal Components (DAPC) will provide a description of clusters using linear combinations of alleles; these combinations are known to have the largest between-groups variance and the smallest within-group variance [36]. Bayesian information criterion (BIC) is provided to describe the numbers of clusters (k).

## 3. Results

**Figure 2:**
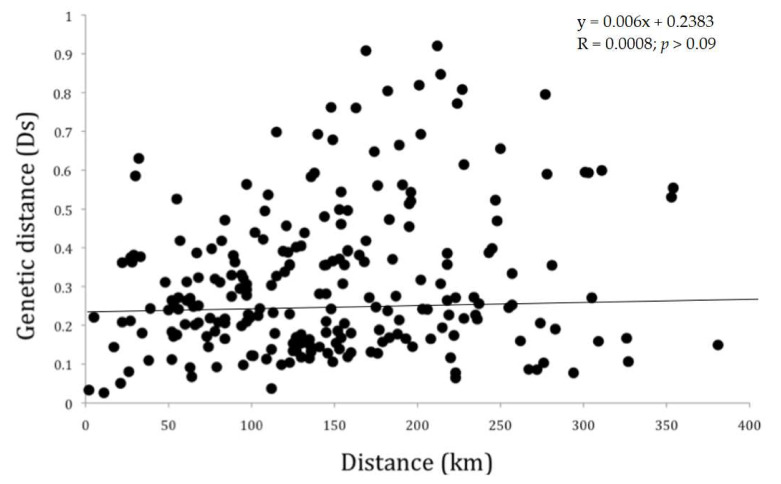
Mantel test based on Ds genetic distance and Euclidean spatial distance (km). There was no relationship between genetic and geographic distance in wild pigs sampled in 24 sites during 2005–2009 in southern Texas, USA.

We genotyped 1258 adult (≥1 year old) wild pigs from 24 sites at 13 microsatellite loci (Table S1). We detected no departures from HWE in populations after Bonferroni correction (Table S1). We found no evidence of linkage disequilibrium. The analysis of genetic and spatial distance revealed no support for isolation by distance pattern (Figure 2). For instance, Ds values were similar between geographically proximate and geographically distant sites (WILL—WT and CAM—CR, respectively). The DR and KRG sites had the least genetic divergence (Ds = 0.026), while AR and WILL had the greatest genetic divergence (Ds = 0.920). Similarly, the AR and WWR sites are located ca. 50 km apart but were genetically divergent (Ds = 0.239); likewise, pairwise Ds values of 0.03, 0.59, and 0.14 were observed at geographic distances of 2 km, 138 km, and 381 km, respectively. The Mantel test results revealed no statistically significant relationship between genetic and spatial distance (Figure 2). The slope of the linear model was nearly 0 (y = 0.0006x + 0.2382) and the matrix correlation was not significantly different from 0.0 (Spearman Rank correlation coefficient, *p* > 0.09; Figure 2). 

At a broad scale, wild pig populations displayed a moderate degree of genetic structure (F_ST_ = 0.11 ± 0.005). The F_ST_ pairwise comparisons among the 24 sites ranged from 0.030 to 0.312, with 236 pairwise comparisons statistically different from 0.0, Table S2). Overall, the F_ST_ values generally corresponded to the Ds values. The WILL and AR were the most genetically divergent sites (F_ST_ = 0.312; Table S2), while the SAD and DU (F_ST_ = 0.032; Table S2) and WILL and WT displayed the greatest genetic similarity compared to the rest of the study sites (F_ST_ = 0.030). 

**Figure 3:**
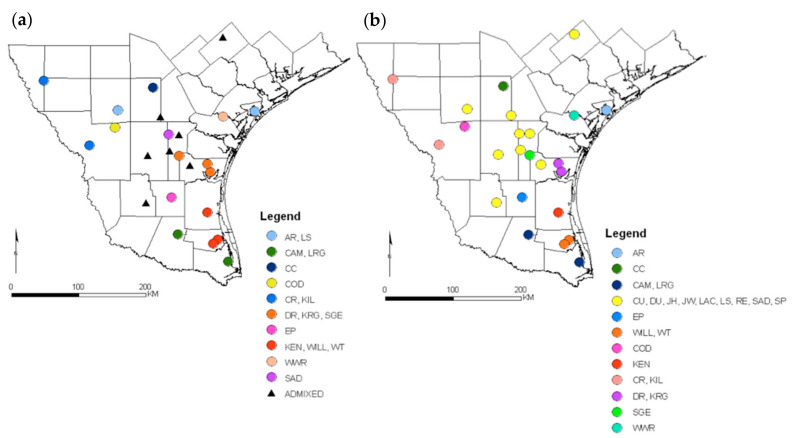
Geographic distribution of genetic clusters based on the best partition generated in the Bayesian clustering algorithm, Structure 2.2 (**a**) (assuming K = 10) and in the Bayesian spatial clustering algorithm BAPS 4.2 (**b**) (assuming K = 12) from wild pigs collected in 24 sites during 2005–2009 in southern Texas, USA.

The posterior probability for number of discrete genetic clusters from Structure was close to 1.0 for K = 10 genetic clusters; the ΔK method of Evanno et al. [30] (Table S3) also supported K = 10 discrete genetic clusters (Figure S1). The sampling sites grouped into genetic clusters were broadly distributed, discrete, or highly admixed (Figure 3). Sites AR-LS, CAM-LRG, CR-KIL, DR-KRG-SGE, and KEN-WILL-WT were partitioned together, whereas sites CC, COD, EP, WWR, and SAD appeared to represent discrete clusters (Figure 3). The CU-RE-JW-DI-LAC-SP-JH sites had a high degree of admixture. 

**Figure 4:**
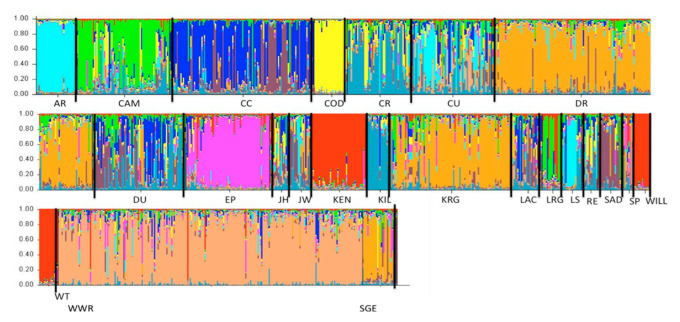
Wild pigs sampled at 24 sites during 2005–2009 in southern Texas, USA. Each individual is represented by a vertical line, which is partitioned into colored segments that represent the individual’s estimated membership fractions in the K = 10 genetic clusters derived from the Bayesian clustering algorithm Structure 2.2. Sampling sites are labeled below the figure. Aransas National Wildlife Refuge (AR), Cameron County (CAM), Choke Canyon State Park (CC), Kubala’s Ranch (COD), Comanche Ranch (CR), Cuero County (CU), Don Ricardo pasture, Laureles Division of King Ranch (DR), Duval County (DU), El Pintor Ranch (EP), Jim Hogg County (JH), Jim Wells County (JW), Kenedy Ranch (KEN), Killam Ranch (KIL), Gallito pature, Laureles Division of King Ranch (KRG), the Texas A&M Extension Service La Copita Research Area (LAC), Lower Rio Grande Valley National Wildlife Refuge (LRG), La Salle County (LS), Rancho Escondido (RE), Santa Gertrudis division of King Ranch (SGE), San Diego County (SAD), South Pasture-Texas A&M-Kingsville (SP), Willacy County (WILL), Wilbarger Tract, Lower Rio Grande Valley National Wildlife Refuge (WT), and Rob and Bessie Welder Wildlife Refuge (WWR).

The spatially explicit clustering from BAPS produced a probability of >0.99 for 12 genetic clusters in the region. Both Structure and BAPS were consistent with the Mantel test in finding no support for a relationship between genetic and geographic distance, as genetically distinct clusters occurred in geographically proximate sites, while sampling sites in the same genetic cluster were broadly dispersed geographically. The BAPS results indicated that the CAM-LRG, CU-DU-JH-JW-LAC-LS-RE-SAD-SP, WILL-WT, CR-KILL sites, and the DR-KRG sites represented five genetic clusters. In contrast, the AR, CC, EP, COD, KEN, SGE, and WWR sites represented genetically discrete clusters. Only four sites out of 24 (if the admixed sites are considered as a single cluster), LS, SGE, KEN, and SAD, differed from the Structure 2.2 partition (Figure 4). The LS and SAD sites became part of the admixed group, while SGE and KEN were differentiated as discrete clusters (Figure 4). The Kullback–Leibler neighbor-joining tree illustrates genetic differentiation among the 12 clusters delineated using the BAPS algorithm (Figure S2) and offers a further indication of the similarity between the Structure and BAPS results. Most differences between the two algorithms corresponded to populations with low Kullback–Leibler divergence, such as KEN and the WIL-WIT cluster, SGE and the DR-KR cluster, and the AR and LS clusters (Figure S2). No clusters were identified using the PCA (Figure S3), similarly no clusters were identified using DAPC, BIC decreased with the number of clusters (k) and not breakage on the line was detected (Figure S4).

## 4. Discussion

Wild pig populations were structured genetically across southern Texas, indicating that some populations were isolated from one another. However, many individuals and local populations were admixed, which indicates that multiple introductions and artificial movement of individuals has occurred. Furthermore, the genetic clusters were not responding to isolation by geographic distance; some members of the same cluster were widely dispersed. A degree of admixture in populations of wild pig is not surprising. Wild pigs have been present in Texas for more than 300 years and are derived from a mixture of escaped domestics and wild pigs from Eurasia released for hunting [37]. However, our genetic data suggest the demographic history of wild pigs appears more complicated than anticipated. 

The two Bayesian approaches implemented here produced similar results, with minor differences in number of clusters. Whereas the Structure analysis supported 10 clusters as the best partition given the data, BAPS identified 12 clusters when spatial locations were considered. Structure was inconclusive assigning 7 sampling sites to a discrete genetic cluster due to the high degree of admixture. The BAPS algorithm clustered the 7 admixed populations as a single genetic cluster. The discrepancy, although slight, may be partly due to different method of clustering (e.g., minimizing HWE and linkage vs. allele frequency divergence). This incongruence between Structure and BAPS has been frequently reported, and BAPS tends to increase the number of clusters [18,38,39,40,41]. However, admixed individuals from geographically dispersed sites may have complicated the BAPS analysis, as the spatial data are used as priors in the clustering analysis. The seven admixed sites are located in an area where large contiguous properties are rare; thus, illegal translocation may be common in the area, exchanging pigs from one property with others. Wild pig populations in Florida showed a similar pattern of high level of admixture suggesting human-mediated dispersal [42]. The PCA and DAPC reassured the presence of admixed individuals (Figures S3 and S4). Our data resembled genetic structure in populations of large mammals that were restored through the use of disparate genetic stocks [43] where genetically similar populations are widely dispersed. However, reports of multiple introductions and artificial movements of invasive species have appeared in the recent literature, suggesting that admixture in populations of invasive species may become increasingly common [18,42,44,45]. We cannot determine the effect of historical admixture, but the genetic data suggest many translocations have occurred in the recent past and may be ongoing. Researchers had reported historical admixed ancestry on wild pigs introduced in the United States with Western heritage breeds and European wild boar of the highest input [46].

Unfortunately for practitioners tasked with managing wild pigs, we identified few barriers to movement other than urban areas and expansive agriculture. The conversion of additional rangeland to crops that might form a suitable barrier is not recommended and urban development tends to radiate outward from existing sites. Therefore, wild pig damage will likely continue or intensify in the foreseeable future in the region. Lack of substantial geographic barriers to movements indicates that achieving long-term wild pig control may be difficult due to large geographic extent of populations and ability to re-colonize managed areas from nearby viable populations. Texas has very little public land (<3%), and current management efforts aimed at alleviating local damage are conducted at relatively small spatial scales, from a few hundred to several thousand ha [47,48]. The formation of management cooperatives among landowners may be necessary to manage spatially extensive areas of southern Texas. 

Large-scale management of wild pigs may be necessary to achieve control in extreme circumstances, such as a foreign animal disease outbreak (i.e., foot and mouth disease). New management tools will be needed for such contingencies, lending support for ongoing research on toxicants [47,49,50], fertility control agents [51,52,53], vaccines [54], and oral delivery systems for these pharmaceuticals [55,56,57,58]. If large-scale control efforts are necessary, the integration of adaptive management and fine-scale spatial data may aid in control efforts [59].

Translocations had a persistent effect on genetic structure. Therefore, it will be difficult to use molecular tools to verify point of origin for illegal translocations or disease management [60] because similar genetic stocks are present in multiple areas. Furthermore, wild pigs have expanded their geographic range in Texas and elsewhere during the past two decades. The rapid expansion of wild pigs may be due more to human-mediated transport than to natural dispersal, as observed in other invasive species [42,44]. Each species and introduction have a unique invasion history that may result in different demographic outcomes [61]. Nevertheless, the easiest means of preventing colonization into new areas will be to halt translocations and other human-mediated transport [62]. This will require enforcement of existing regulations and greater public awareness. 

## 5. Conclusions

These results are an attempt to understand the genetic structure and movement patterns of feral pigs in southern Texas. We expect that this study will have a significant impact increasing the efficiency of control methods and helping define the geographic area over which control methods should be conducted to achieve long-term results. However, the degree of human-mediated admixture, involving individuals from disparate populations, may have complicated the genetic analyses. The admixed sites are located in an area where large contiguous properties are rare; thus, illegal translocation may be common in the area, exchanging pigs from one property to others. It appears that the Structure partition may be a more realistic and coherent partition due to highly degree of admixture and landscape characteristics among the admixed sites. Differences in USA wild pig populations compared to Australia and Europe include historical and ongoing undocumented translocations and water lack or availability distributed along the landscape for livestock. These two factors contribute to a high degree of admixture among wild pigs in USA. Management cooperatives may be necessary to manage spatially extensive areas of southern Texas. Facing the difficulty of large-scale wildlife management for diseases, damages, and invasiveness, wildlife management personnel may be able to improve the efficiency and effectiveness of large-scale management if they can consider terrain features that affect animal movements and population structuring. 

## Data Availability

The data presented in this study are available on request from the corresponding author.

## Supplementary Materials

The following are available online at https://www.mdpi.com/2076-2615/11/1/168/s1, Table S1: Observed (HObs) and expected heterozygosity (HExp), number of alleles (n) at each of the 13 microsatellite DNA loci amplified in wild populations in 24 study sites during 2005–2009 in southern Texas, USA. All loci are in Hardy–Weinberg equilibrium, Table S2: Nei’s (1972) Ds genetic pairwise genetic distance (upper matrix) and Weir and Cockerham’s (1984) FST pairwise 5 comparisons among 24 study sites (lower matrix) during 2005–2009 in southern Texas, USA. The RE and SP sites did not have enough sample size to perform the analysis. Asterisks indicate FST values that are statistically different from 0.0, Table S3: Estimated posterior probability and their variance based on Bayes’ Rule for the 11 best partition for the number of populations in Structure 2.2 [29]. Based on wild pigs sampled in 24 sites during 2005–2009 in southern Texas, USA. Model choice criterion (Ln P(D)); estimated model log-likelihood (Log P(K/X)); variance of the model choice criterion (Var(Ln P(D)), Figure S1: Second order rate of change of the likelihood distribution [30] for the best 18 partition of the genetic clusters generated in the Bayesian clustering algorithm, Structure 2.2, based on samples from 24 sites collected during 2005–2009 in southern Texas, USA. The 2nd order rate of change of the likelihood distribution corresponds to K = 10 discrete genetic clusters, Figure S2: Neighbor-joining unrooted tree for the Kullback–Leibler divergence matrix produced by Structure 2.2 (a) and BAPS 4.2 (b). The Kullback–Leibler can be used as a genetic distance matrix among 10 clusters produced by Structure 2.2 and 12 clusters produced by BAPS 4.2. The Bayesian clustering algorithms are based on wild pig samples collected in 24 sites during 2005–2009 in southern Texas, USA, Figure S3: Wild pigs sampled at 24 sites during 2005–2009 in southern Texas, USA. 44 Each individual is represented by dot, and each color represents the individual’s collection site. PC1 explains 53% of the variance and PC2 explains 50% of the variance, Figure S4: Bayesian information criterion (BIC) describing the numbers of clusters 60 (k) for wild pigs sampled at 24 sites during 2005–2009 in southern Texas, USA. No clusters were identified using DAPC, BIC decrease with the number of clusters (k), and no breakage on the line was detected.
