# Supplementary material for: Genetic Population Structure of Wild Pigs in Southern Texas
Source: Animals (Basel). 2021 Jan 12;11(1):168. doi: 10.3390/ani11010168 (PMC7828165; doi:10.3390/ani11010168)
Supplement: Supplementary file 1 [file animals-11-00168-s001.pdf]

animals MDPI

Table S1 Observed (H_{Obs}) and expected heterozygosity (H_{Exp}), number of alleles (n) at each of the 13 microsatellite DNA loci amplified in wild pig populations in 24 study sites during 2005-2009 in southern Texas, USA. All loci are in Hardy-Weinberg equilibrium.

Locus	AR (n=33)			CAM (n=75)			CC (n=117)			COD (n=27)			CR (n=51)		
	n	Hobs	Hexp	n	Hobs	Hexp	n	Hobs	Hexp	n	Hobs	Hexp	n	Hobs	Hobs
S0002	4	0.667	0.624	6	0.734	0.761	5	0.552	0.693	3	0.815	0.643	8	0.643	0.748
S0026	3	0.656	0.516	3	0.425	0.412	4	0.543	0.546	4	0.556	0.476	3	0.592	0.565
S0068	2	0.133	0.235	7	0.536	0.662	7	0.627	0.686	3	0.654	0.486	8	0.432	0.695
S0090	3	0.300	0.267	4	0.413	0.398	4	0.585	0.547	2	0.444	0.352	3	0.308	0.369
S0155	3	0.615	0.577	5	0.528	0.695	6	0.558	0.606	4	0.593	0.597	7	0.522	0.532
S0226	4	0.594	0.682	5	0.616	0.669	5	0.598	0.581	3	0.346	0.429	4	0.612	0.588
SW122	3	0.563	0.597	6	0.581	0.675	6	0.803	0.802	3	0.741	0.676	6	0.588	0.634
SW240	5	0.484	0.605	6	0.649	0.661	7	0.823	0.820	4	0.731	0.669	5	0.636	0.736
SW632	5	0.871	0.712	5	0.720	0.749	7	0.759	0.762	2	0.481	0.440	7	0.673	0.633
SW857	4	0.414	0.476	4	0.600	0.661	5	0.397	0.537	3	0.704	0.586	4	0.465	0.574
SW911	4	0.484	0.507	4	0.554	0.542	4	0.496	0.469	3	0.333	0.293	3	0.686	0.577
SW936	5	0.818	0.740	7	0.811	0.802	6	0.764	0.749	3	0.815	0.677	7	0.735	0.775
SW951	3	0.406	0.335	3	0.286	0.279	6	0.467	0.430	4	0.407	0.400	4	0.313	0.277

Table S1. Continued.

	CU (n=66)			DR (n=142)			DU (n=76)			EP (n=68)			JH (n=16)		
Locus	n	Hobs	Hexp	n	Hobs	Hexp	n	Hobs	Hexp	n	Hobs	Hexp	n	Hobs	Hexp
S0002	8	0.547	0.750	7	0.690	0.665	8	0.617	0.783	5	0.821	0.736	5	0.643	0.643
S0026	5	0.400	0.432	4	0.384	0.407	3	0.493	0.529	4	0.574	0.606	4	0.625	0.607
S0068	8	0.408	0.578	10	0.788	0.866	9	0.644	0.662	8	0.869	0.792	4	0.364	0.403
S0090	4	0.396	0.443	3	0.315	0.277	3	0.297	0.266	3	0.516	0.509	3	0.286	0.474
S0155	7	0.600	0.733	6	0.780	0.752	5	0.704	0.618	3	0.625	0.607	5	0.538	0.757
S0226	6	0.600	0.701	6	0.677	0.724	4	0.568	0.555	6	0.773	0.794	6	0.625	0.774

SW122	8	0.734	0.760	7	0.733	0.686	7	0.797	0.732	5	0.552	0.591	7	0.938	0.802
SW240	8	0.636	0.825	7	0.670	0.724	5	0.647	0.723	6	0.745	0.767	7	0.538	0.745
SW632	8	0.742	0.771	7	0.759	0.762	7	0.797	0.793	5	0.706	0.744	5	0.625	0.714
SW857	5	0.484	0.516	4	0.661	0.662	5	0.486	0.661	5	0.500	0.718	2	0.273	0.247
SW911	6	0.556	0.574	3	0.538	0.534	4	0.356	0.426	5	0.565	0.585	2	0.313	0.466
SW936	8	0.741	0.824	8	0.790	0.777	8	0.795	0.808	7	0.754	0.779	6	0.600	0.724
SW951	5	0.367	0.336	4	0.459	0.464	5	0.307	0.328	4	0.537	0.471	3	0.533	0.600

Table S1. Continued.

	JW (n=17)			KEN (n=45)			KIL (n=20)			KRG (n=97)			LAC (n=26)		
Locus	n	Hobs	Hexp	n	Hobs	Hexp	n	Hobs	Hexp	n	Hobs	Hexp	n	Hobs	Hexp
S0002	4	0.429	0.396	5	$0.738	$0.721	7	0.875	0.744	8	0.605	0.662	6	0.722	0.702
S0026	2	0.353	0.499	4	$0.467	$0.621	3	0.550	0.606	4	0.629	0.610	4	0.667	0.671
S0068	7	0.583	0.779	7	$0.273	$0.410	2	0.125	0.125	9	0.859	0.848	8	0.846	0.868
S0090	2	0.091	0.091	4	$0.444	$0.452	2	0.438	0.353	3	0.300	0.341	3	0.867	0.683
S0155	5	0.857	0.802	5	$0.622	$0.583	4	0.556	0.649	5	0.707	0.753	3	0.682	0.585
S0226	3	0.625	0.655	5	$0.778	$0.721	3	0.211	0.280	5	0.652	0.699	4	0.391	0.691
SW122	6	0.765	0.658	2	$0.356	$0.296	5	0.800	0.759	7	0.717	0.767	7	0.762	0.782
SW240	6	0.800	0.811	6	$0.422	$0.711	5	0.706	0.706	7	0.718	0.749	7	0.727	0.766
SW632	5	0.857	0.804	6	$0.636	$0.711	4	0.650	0.642	6	0.653	0.647	5	0.684	0.750
SW857	2	0.250	0.536	5	$0.500	$0.679	3	0.722	0.660	5	0.702	0.635	4	0.682	0.720
SW911	4	0.875	0.720	3	$0.050	$0.097	4	0.600	0.499	4	0.584	0.548	4	0.500	0.424
SW936	6	0.875	0.841	7	$0.909	$0.807	5	0.737	0.747	8	0.708	0.721	5	0.792	0.776
SW951	4	0.438	0.466	3	$0.349	$0.298	3	0.700	0.621	4	0.333	0.462	3	0.227	0.212

Table S1. Continued.

	LRG (n=15)			LS (n=17)			RE (n=13)			SAD (n=18)			SP (n=10)		
Locus	n	Hobs	Hexp	n	Hobs	Hexp	n	Hobs	Hexp	n	Hobs	Hexp	n	Hobs	Hexp
S0002	4	0.467	0.634	6	0.600	0.782	4	0.800	0.700	5	0.684	0.760	4	0.667	0.647
S0026	3	0.600	0.503	3	0.824	0.611	4	0.417	0.482	3	0.158	0.243	3	0.400	0.611

S0068	5	0.833	0.786	5	0.412	0.554	3	0.364	0.325	4	0.833	0.743	7	1.000	0.867
S0090	3	0.500	0.405	2	0.125	0.121	3	0.455	0.498	4	0.474	0.467	3	0.333	0.307
S0155	4	0.786	0.706	4	0.706	0.754	5	0.583	0.688	4	0.833	0.703	3	0.500	0.484
S0226	3	0.286	0.553	5	0.882	0.729	4	0.545	0.558	5	0.737	0.639	6	0.900	0.821
SW122	4	0.400	0.356	4	0.824	0.651	5	0.500	0.699	5	0.737	0.704	4	0.700	0.726
SW240	4	0.583	0.692	5	0.529	0.661	3	0.636	0.662	3	0.765	0.656	7	0.875	0.792
SW632	4	0.643	0.601	6	0.750	0.770	4	0.636	0.636	7	0.842	0.839	5	1.000	0.817
SW857	3	0.250	0.562	2	0.133	0.129	4	0.700	0.726	5	0.526	0.653	2	0.000	0.429
SW911	2	0.467	0.508	3	0.588	0.540	2	0.500	0.391	3	0.579	0.568	3	0.400	0.468
SW936	6	0.714	0.767	6	0.824	0.793	7	0.750	0.826	6	0.526	0.691	6	0.800	0.811
SW951	3	0.600	0.476	4	0.765	0.629	3	0.583	0.489	3	0.158	0.152	3	0.400	0.353

Table S2 Nei's (1972) Ds genetic pairwise genetic distance (upper matrix) and Weir and Cockerham's (1984) F_{ST} pairwise comparisons among 24 study sites (lower matrix) during 2005-2009 in southern Texas, USA. The RE and SP sites did not have enough sample size to perform the analysis. Asterisks indicate F_{ST} values that are statistically different from 0.0

CC	COD	CR	CU	DR	DU	EP	JH	JW	KEN	KIL	KRG	LAC	LRG	LS	RE	SAD	SP	WILL	WT	WWR	SGE
0.307	0.194	0.167	0.098	0.338	0.264	0.473	0.176	0.210	0.908	0.227	0.327	0.481	0.064	0.241	-	0.593	-	0.439	0.920	0.847	0.240
0.103	0.246	0.149	0.106	0.132	0.077	0.134	0.271	0.188	0.311	0.218	0.128	0.237	0.144	0.086	-	0.242	-	0.106	0.381	0.363	0.307
	0.165	0.131	0.138	0.182	0.037	0.180	0.312	0.122	0.455	0.169	0.164	0.097	0.174	0.091	-	0.171	-	0.133	0.523	0.470	0.303
0.108*		0.154	0.145	0.371	0.243	0.405	0.281	0.294	0.804	0.311	0.356	0.363	0.177	0.212	-	0.418	-	0.401	0.808	0.614	0.381
0.064*	0.111*		0.086	0.190	0.119	0.334	0.386	0.271	0.599	0.168	0.206	0.227	0.159	0.153	-	0.165	-	0.253	0.531	0.554	0.355
0.049*	0.099*	0.047*		0.166	0.116	0.256	0.171	0.157	0.655	0.216	0.168	0.247	0.077	0.127	-	0.356	-	0.214	0.595	0.593	0.179
0.089*	0.161*	0.084*	0.080*		0.115	0.248	0.418	0.203	0.418	0.366	0.026	0.261	0.243	0.130	-	0.320	-	0.080	0.495	0.537	0.472
0.025*	0.116*	0.054*	0.046*	0.077*		0.220	0.271	0.092	0.461	0.144	0.118	0.184	0.139	0.067	-	0.208	-	0.113	0.514	0.520	0.364
0.085*	0.141*	0.117*	0.091*	0.121*	0.092*		0.317	0.264	0.242	0.422	0.251	0.201	0.270	0.242	-	0.329	-	0.112	0.292	0.228	0.281
0.077*	0.136*	0.121*	0.057*	0.138*	0.083*	0.110*		0.144	0.772	0.275	0.393	0.583	0.160	0.229	-	0.698	-	0.391	0.795	0.590	0.225
0.089*	0.154*	0.131*	0.080*	0.093*	0.078*	0.109*	0.089*		0.563	0.199	0.183	0.221	0.104	0.121	-	0.374	-	0.180	0.762	0.678	0.306
0.160*	0.244*	0.183*	0.190*	0.189*	0.167*	0.107*	0.200*	0.225		0.760	0.387	0.440	0.526	0.543	-	0.357	-	0.207	0.264	0.246	0.693
0.088*	0.158*	0.103	0.103	0.159*	0.081*	0.153*	0.087	0.132	0.258		0.355	0.330	0.205	0.218	-	0.277	-	0.355	0.819	0.693	0.370
0.078*	0.170*	0.077*	0.071*	0.019*	0.066*	0.115*	0.124*	0.095	0.167*	0.154*		0.176	0.233	0.154	-	0.323	-	0.051	0.391	0.457	0.397
0.052*	0.133*	0.085*	0.082*	0.093*	0.056*	0.075*	0.120*	0.089	0.147*	0.152	0.064*		0.362	0.209	-	0.208	-	0.109	0.499	0.544	0.321
0.093*	0.183*	0.123*	0.112*	0.113*	0.092*	0.091*	0.125*	0.140	0.122*	0.168	0.118*	0.105*		0.217	-	0.206	-	0.150	0.387	0.398	0.497
0.079*	0.150*	0.092*	0.032*	0.118*	0.083*	0.109*	0.070*	0.096	0.210*	0.111	0.103*	0.138	0.127*		-	0.377	-	0.271	0.648	0.560	0.275
0.059*	0.110*	0.074*	0.053*	0.109*	0.041*	0.096*	0.070	0.075	0.208*	0.096	0.102*	0.083	0.139	0.095		-	-	-	-	-	-
0.05* 0	0.126*	0.087*	0.068*	0.074*	0.032*	0.104*	0.096*	0.083	0.189	0.136	0.076*	0.061	0.126*	0.132	0.037*		-	0.186	0.630	0.585	0.357
0.065*	0.169*	0.081*	0.083*	0.060*	0.047*	0.067*	0.141*	0.113	0.122	0.159	0.045*	0.035	0.118*	0.143*	0.096*	0.053*		-	-	-	-
0.189*	0.266*	0.201*	0.203*	0.213*	0.198*	0.125*	0.235*	0.272	0.084	0.292	0.186*	0.185	0.161*	0.248*	0.238*	0.238	0.177*		0.033	0.388	0.380
0.179*	0.240*	0.211*	0.199*	0.228*	0.200*	0.114*	0.211*	0.270	0.100	0.265	0.209*	0.200	0.182*	0.229*	0.230*	0.230	0.185*	0.030*		0.033	0.665
0.103*	0.188*	0.133*	0.064*	0.146*	0.124*	0.109*	0.095*	0.121	0.201*	0.167	0.124*	0.119	0.174*	0.091*	0.125*	0.142*	0.149*	0.222*	0.211*		0.562
0.074*	0.185*	0.093*	0.085*	0.052*	0.080*	0.089*	0.124*	0.093	0.157*	0.139	0.054*	0.068	0.107*	0.128*	0.109*	0.075	0.057*	0.202*	0.210*	0.125*	

Table 3. Estimated posterior probability and their variance based on Bayes' Rule for the best partition for the number of populations in Structure 2.2 [29]. Based on wild pigs sampled in 24 sites during 2005-2009 in southern Texas, USA. Model choice criterion (Ln P(D)); estimated model log-likelihood (Log P(K/X)); variance of the model choice criterion (Var[Ln P(D)]).

K	Ln P(D)	Log P(K/X)	Var[LnP(D)]
1	-38959.05	0.000055	47.2981
2	-42260.93	0.000000	2.4000
3	-41053.02	0.000006	23.6559
4	-39885.97	0.000013	7.1853
5	-39451.93	0.000020	5.1850
6	-39220.60	0.000000	38.3806
7	-39017.41	0.000046	90.3912
8	-38705.41	0.000640	98.0858
9	-38510.34	0.000143	48.9844
10	-38358.65	0.900047	29.6011
11	-38200.29	0.000032	18.6862
12	-38085.84	0.000026	37.2553
13	-37989.26	-0.000022	13.6308
14	-37889.39	-0.000019	20.3476
15	-37827.01	-0.000019	99.1972
16	-37754.11	-0.000017	41.2970
17	-37743.35	-0.000016	50.2734
18	-38847.00	0.000093	3225.1237
19	-37683.30	-0.000015	106.8053
20	-37672.07	-0.000015	340.5906
21	-38091.96	-0.000026	1625.6877
22	-38041.73	-0.000024	1651.5910
23	-38317.80	-0.000042	1283.9294
24	-38788.40	-0.000090	1144896.8112

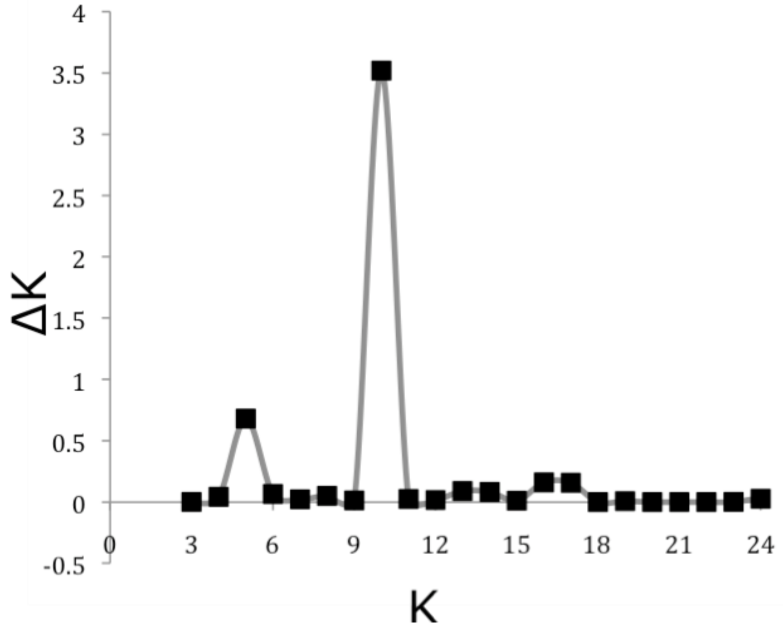

Figure S1. Second order rate of change of the likelihood distribution [30] for the best partition of the genetic clusters generated in the Bayesian clustering algorithm, Structure 2.2, based on samples from 24 sites collected during 2005-2009 in southern Texas, USA. The 2nd order rate of change of the likelihood distribution corresponds to K = 10 discrete genetic clusters.

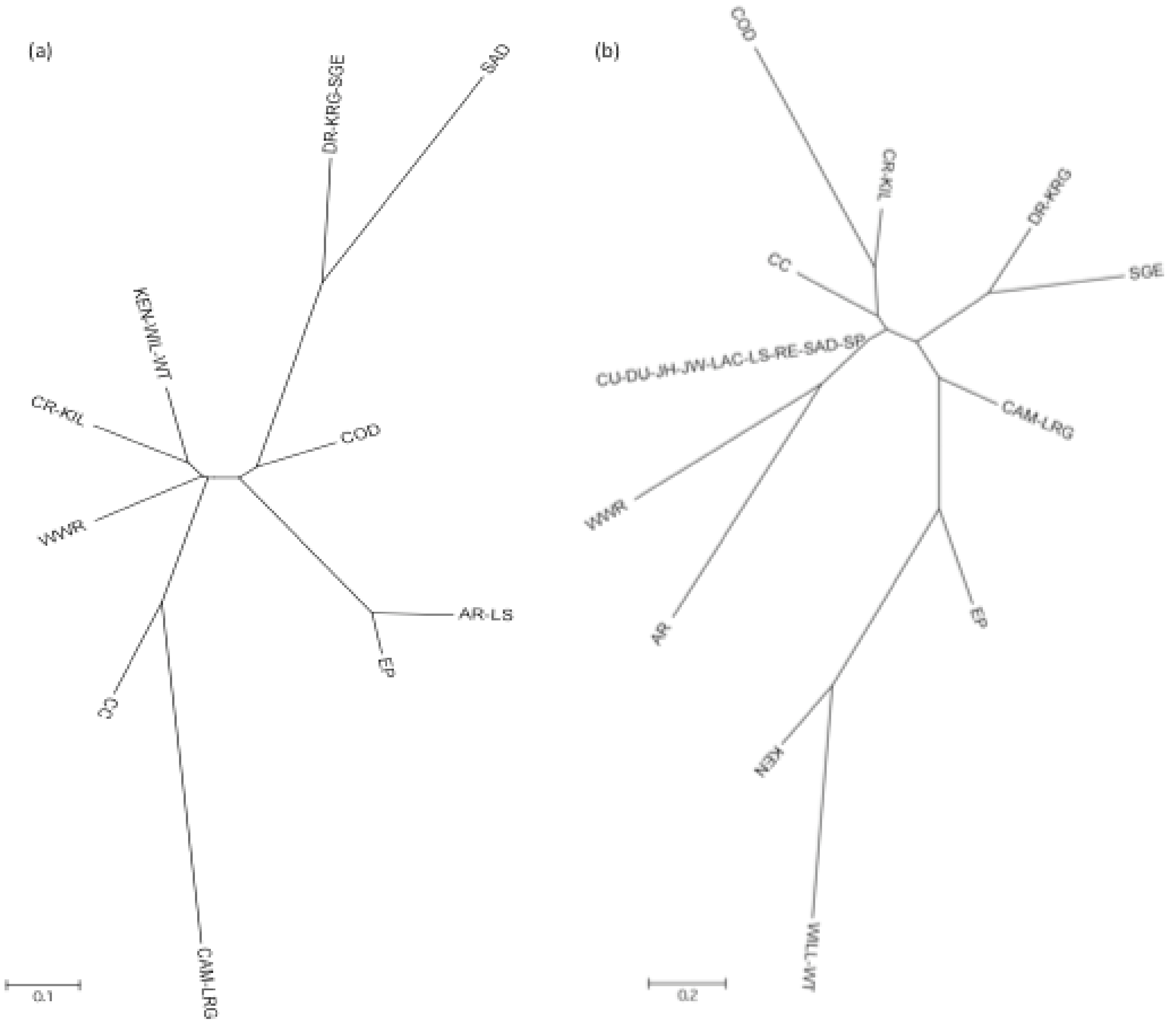

Figure S2 Neighbor-joining unrooted tree for the Kullback-Leibler divergence matrix produced by Structure **2.2** (a) and BAPS 4.2 (b). The Kulback-Leibler can be used as a genetic distance matrix among 10 clusters produced by StrucTable 2. 2 and 12 clusters produced by BAPS 4.2. The Bayesian clustering algorithms are based on wild pig samples collected in 24 sites during 2005-2009 in southern Texas, USA.

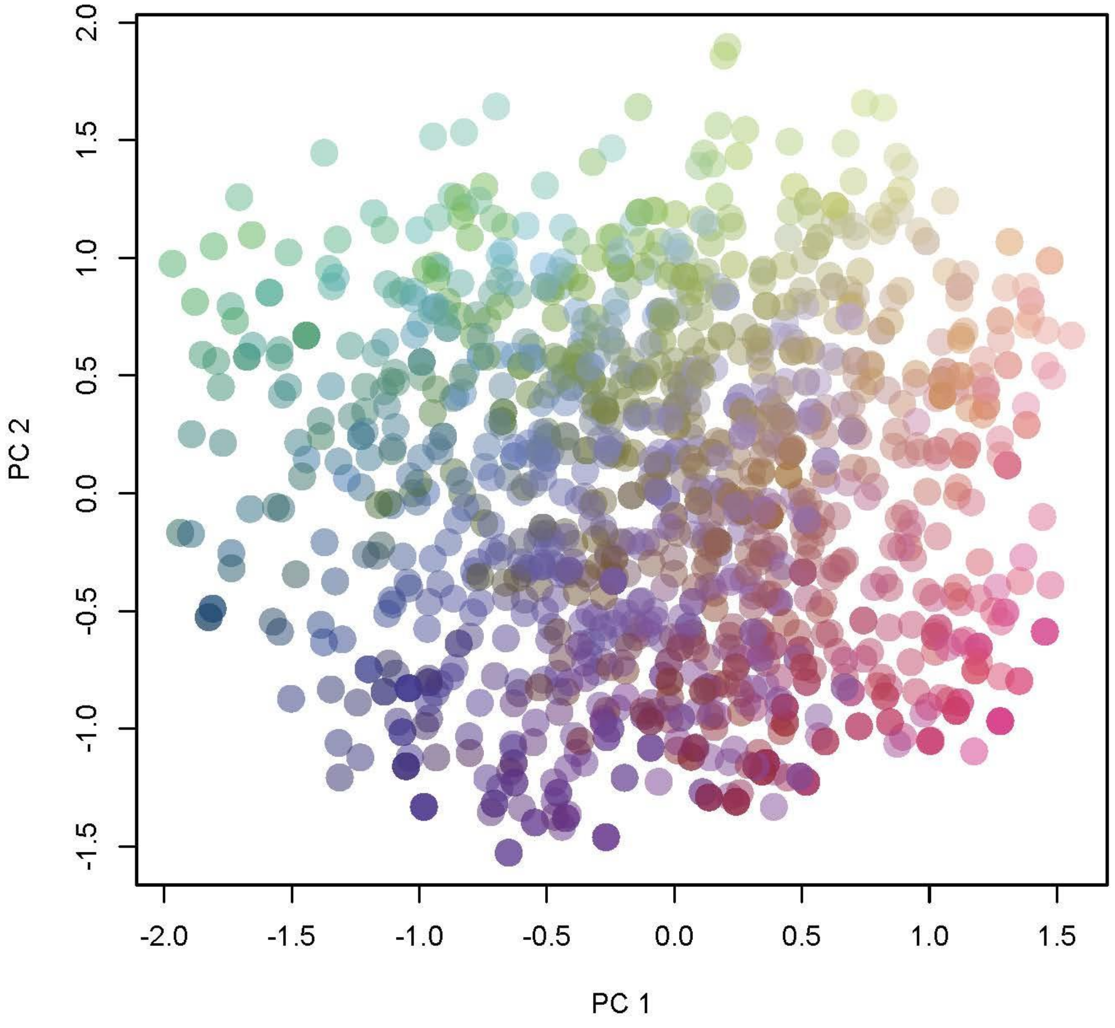

Figure 3. Wild pigs sampled at 24 sites during 2005-2009 in southern Texas, USA. Each individual is represented by dot, each color represents the individual's collection site. PC1 explains 53% of the variance and PC2 explains 50% of the variance.

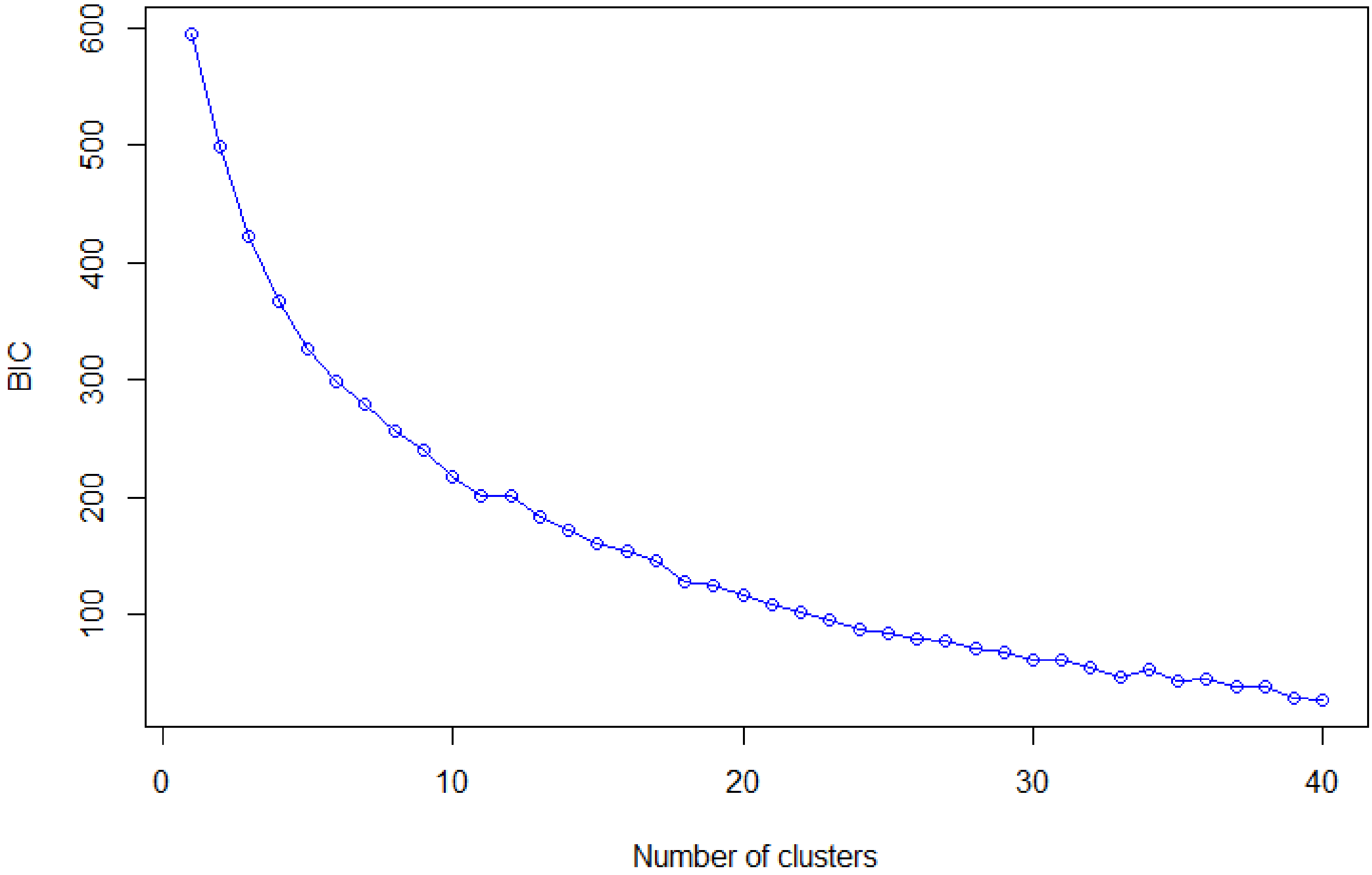

Figure S4. Bayesian information criterion (BIC) describing the numbers of clusters (k) for wild pigs sampled at 24 sites during 2005-2009 in southern Texas, USA. No clusters were identified using DAPC, BIC decrease with the number of clusters (k) and not breakage on the line was detected.
